# Data of RNA-seq transcriptomes in the brain associated with aggression in males of the fish *Betta splendens*

**DOI:** 10.1016/j.dib.2021.107448

**Published:** 2021-10-02

**Authors:** Trieu-Duc Vu, Yuki Iwasaki, Kenshiro Oshima, Ming-Tzu Chiu, Masato Nikaido, Norihiro Okada

**Affiliations:** a School of Pharmacy, Kitasato University, Tokyo, Japan; b Life Sciences and Biotechnology Department, Tokyo Institute of Technology, Tokyo, Japan; c Department of Life Sciences, National Cheng Kung University, Tainan, Taiwan; d Nagahama Institute of Bio-Science and Technology, Nagahama, Japan

**Keywords:** RNA-seq data, *Betta splendens*, Aggression, DEG

## Abstract

Siamese fighting fish *Betta splendens* are notorious for their aggressiveness and males of this fish have been widely used to study aggression. However, an understanding of brain transcriptome signature associated with aggression in the context of male-male interaction in this fish remains to be understood. Herein, RNA-Seq transcriptome data from 37 brains samples collected at different fighting stages are described. These brain samples were collected before fighting (B), during fighting (D20 and D60), and after fighting (A0 and A30). The raw data were analyzed for differential gene expression using edgeR package in *R*. A criterion of FDR cut-off ≤ 0.05 and an absolute fold change (FC) of 0 or greater were used to identify top upregulated and downregulated genes in fighting groups (D20, D60, A0, and A30) relative to non-fighting group (B). The data presented hereafter enable fundamental studies on genes and molecular events mediating aggressive behavior in this fish and will lay a valuable foundation for future research on the aggression of vertebrates*.*

## Specifications Table

**Table:** 

Subject	Biology
Specific subject area	Molecular Biology, Transcriptomics
Type of data	Transcriptomics (RNA-seq)
How data were acquired	High-throughput sequencing (Illumina HiSeq 2500)
Data format	Abundance estimates of raw transcripts (genes) are generated by feature Counts [1]. Normalized gene expression values are presented as median of ratio values generated by the edgeR package [2]. Differential brain gene expression estimates of fighting groups (D20, D60, A0, and A30) verses non-fighting group (B) are calculated using the edgeR package [2].
Parameters for data collection	Whole brains from 37 males of *Betta splendens* collected at different fighting stages namely non-fighting (B, 5 individuals), during fighting for 20 min (D20, 10 individuals), during fighting for 60 min (D60, 10 individuals), immediately after shifting their social status i.e., the winner/loser has emerged (A0, 6 individuals) and 30 min after this shift (A30, 6 individuals) (Fig. S1A).
Description of data collection	RNA was isolated using trizol from whole brains of 37 samples of males *Betta splendens*. Genomics core constructed and sequenced libraries as described in material and methods.
Data source location	Institution: School of Pharmacy, Kitasato University City/Town/Region: Tokyo Country: Japan
Data accessibility	Repository name: DDBJ Data identification numbers: DRA009599 and PRJDB11439 Direct URL to data: https://ddbj.nig.ac.jp/DRASearch/submission?acc=DRA009599
Related research article	Vu Trieu-Duc, Iwasaki Y, Oshima K, Chiu MT, Nikaido M, Okada N. A unique neurogenomic state emerges after aggressive confrontations in males of the fish *Betta splendens*. https://doi.org/10.1016/j.gene.2021.145601

**Value of the Data**

• This data set provides insight into the brain gene expression alteration in males of the fish *B. splendens* in the context of male-male interaction and can further provide insights into other fish species.
• This data set facilitates a comprehensive understanding of aggression at the molecular level among scientists working on fish biology, neuroscientists, and molecular evolutionary biologists.
• This data provides the information of genes associated potentially with long-term memory, hibernation state, and autism spectrum disorder, which will be valuable resources for future study in these respective fields.

## 1 Data Description

To address how a complex social behavior such as aggression is influenced both by genetic and environmental factors [3,4], we identified differentially expressed genes using the RNA sequencing approach [5], which was applied to the male *Betta splendens* collected from different fighting stages namely non-fighting (B), during fighting (D20 and D60), and after fighting (A0 and A30). The results of sequence quality assessment for all the samples are summarized in Table 1 as the number of raw reads, mapped reads, and unique mapped reads (reads that matched the reference genome in only one position). Two types of sequencing were used including single-ended and pair-ended sequencing with different sequence lengths 51, 101, or 126 base pairs (bp). As for pair-ended sequencing, the average raw reads, mapped reads, and unique mapped reads are 39,407,972; 31,888,554; and 30,580,374, respectively. As for single-ended sequencing, the average raw reads, mapped reads, and unique mapped reads are 25,957,661; 21,801,529; and 20,823,889, respectively. The mapping rates for pair-ended and single-ended and sequencing are 79.6% and 79.0%, respectively. Given that the samples had undergone two different sequencing methods, we examined the possibility of whether it led to any biases in the data using multidimensional scaling (MDS) plot. The MDS plot, which was color-coded based on the sequencing method, revealed that the two methods resulted in slight or no biases as all samples were clustered together (Fig. S1B).

**Table 1:** Overview of basic quality of read counts.

Sample	Seq type	Seq length	Raw reads	Clean reads	Rate (%)	Mapped reads	Rate (%)	Unique mapped reads	Rate (%)
B1	PE	126	51,346,604	49,975,204	97.3	40,530,315	81.1	39,238,880	78.5
B2	PE	126	49,140,932	47,916,476	97.5	38,745,629	80.9	37,669,719	78.6
B3	PE	126	48,208,708	46,916,119	97.3	36,413,547	77.7	35,289,963	75.2
D2-11	PE	51	28,992,195	28,483,456	98.2	24,156,876	84.8	22,948,699	80.6
D2-12	PE	51	26,365,612	25,921,898	98.3	20,850,543	80.4	19,634,782	75.7
D2-31	PE	51	31,124,911	30,586,422	98.3	26,241,295	85.8	24,714,267	80.8
D2-32	PE	51	34,611,736	34,028,689	98.3	27,481,148	80.8	25,984,663	76.4
D6-11	PE	126	53,546,536	52,057,680	97.2	42,645,353	81.9	41,162,445	79.1
D6-12	PE	126	53,478,206	52,015,637	97.3	41,457,489	79.7	40,142,687	77.2
D6-21	PE	51	35,483,598	34,884,741	98.3	30,695,163	88.0	29,515,535	84.6
D6-22	PE	51	30,019,262	29,511,844	98.3	25,765,839	87.3	24,528,309	83.1
D6-31	PE	51	37,746,931	37,101,295	98.3	32,767,131	88.3	31,295,160	84.4
D6-32	PE	51	32,238,404	31,699,155	98.3	26,800,877	84.5	25,419,756	80.2

**Ave.**	**PE**		**39,407,972**	**38,546,047**	**97.9**	**31,888,554**	**83.2**	**30,580,374**	**79.6**

W0-1	SE	51	64,343,958	63,898,488	99.3	56,988,137	89.2	54505410	85.3
L0-1	SE	51	67,315,174	66,859,008	99.3	60,563,183	90.7	58093792	86.9
W0-3	SE	51	64,647,278	63,703,066	98.5	56,587,993	88.8	54083903	84.9
L0-3	SE	51	54,707,080	53,860,915	98.5	48,927,544	90.8	46805135	86.9
D2-21	SE	101	16,039,586	15,822,999	98.6	12,206,988	77.1	11,686,243	73.9
D2-22	SE	101	14,104,639	13,927,499	98.7	11,072,535	79.5	10,494,595	75.4
D2-41	SE	101	14,150,818	13,974,411	98.8	11,452,056	82.0	10,981,673	78.6
D2-42	SE	101	13,862,521	13,680,270	98.7	11,283,917	82.5	10,770,098	78.7
D2-51	SE	101	16,295,421	16,086,482	98.7	12,366,775	76.9	11,803,101	73.4
D2-52	SE	101	13,083,352	12,915,583	98.7	10,159,827	78.7	9,705,272	75.1
D6-41	SE	101	15,146,512	14,950,508	98.7	12,507,414	83.7	12,055,255	80.6
D6-42	SE	101	14,901,370	14,702,373	98.7	11,805,871	80.3	11,276,207	76.7
D6-51	SE	101	15,386,535	15,195,196	98.8	12,312,109	81.0	11,794,758	77.6
D6-52	SE	101	16,529,217	16,326,872	98.8	12,853,298	78.7	12,226,599	74.9
B4	SE	101	13,298,709	13,126,356	98.7	10,711,206	81.6	10,190,337	76.7
B5	SE	101	13,772,260	13,576,062	98.6	10,464,211	77.1	10,009,146	72.8
W0-2	SE	51	21,826,538	21,700,996	99.4	17,791,275	82.6	18,463,075	77.9
L0-2	SE	51	24,069,792	21,535,664	89.5	18,056,337	76.2	15,548,749	72.2
W30-1	SE	51	21,418,881	21,155,443	98.8	17,445,194	82.5	16,564,711	78.3
L30-1	SE	51	21,487,398	21,213,396	98.7	16,916,524	79.7	16,016,113	75.5
L30-3	SE	51	24,996,115	24,653,997	98.6	21,711,050	88.1	20,758,665	84.2
W30-3	SE	51	24,266,909	23,959,140	98.7	20,826,896	86.9	19,886,086	83
L30-2	SE	51	28,928,598	28,556,736	98.7	24,895,170	87.2	23,816,317	83.4
W30-2	SE	51	28,405,212	27,972,457	98.5	23,331,186	83.4	22,238,103	79.5

**Ave.**	**SE**		**25,957,661**	**25,556,413**	**98.4**	**21,801,529**	**83.0**	**20,823,889**	**79.0**

**PE**: paired-ended sequencing; **SE**: single-ended sequencing; W0 & L0 belongs to the A0 group, W30 & L30 belongs to the A30 group.

Table 2 shows the number of differentially expressed genes (DEGs) between fighting groups (D20, D60, A0, and A30) relative to the non-fighting group (B) using a criterion of FDR cut-off < 0.05. In this analysis, normalization of differential gene expression is required to obtain more objective values because the number of mapped reads varies with the length of a gene. In doing so, the trimmed mean of M values (TMM) method was implemented using edgR package in *R*. The gene ID, *p*-value, FDR-value, logFC, etc., for each section can be seen in Table S2 in the co-published article.

**Table 2:** Overview of differentially expressed genes (DEGs).

Group 1 (G1)	Group 2 (G2)	No. of DEGs*	Up-regulated⁎⁎	Down-regulated⁎⁎⁎
*B*	D20	1,318	1,148	170
*B*	D60	3,912	3,002	910
*B*	A0	4,756	2,941	1,815
*B*	A30	2,480	1,640	840

⁎ When the FDR-value of the expression level in the same gene between the two groups was less than 0.05, the difference was significant and the number was indicated.

⁎⁎ The expression of gene higher in G2 group than G1 group.

⁎⁎⁎ The expression of gene lower in G2 group than G1 group.

Table 3 shows the enrichment of the biological process of the DEG list for the D20, D60, A0, A30 relative to the *B* group. Detailed GO IDs, gene names, descriptions, etc., are provided in Table S4 in the co-published article.

**Table 3:** Overview of gene ontology.

Comparisons		up-regulated		down-regulated	
Group 1 (G1)	Group 1 (G2)	No. of DEG*	No. of BP⁎⁎	No. of DEG*	No. of BP⁎⁎
*B*	D20	387	7	37	7
*B*	D60	727	14	35	8
*B*	A0	642	12	204	1
*B*	A30	395	7	140	5

⁎ Among the genes with significant difference between two groups, the number of gene which FDR is less than 0.05 & |logFC| > 2 is indicated.

⁎⁎ BP: Biological process. The terms with a p-value of less than 0.01 are indicated.

## 2 Experimental Design, Materials and Methods

### 2.1 Experimental design

#### 2.1.1 Sample collection

**Fig. 1:**
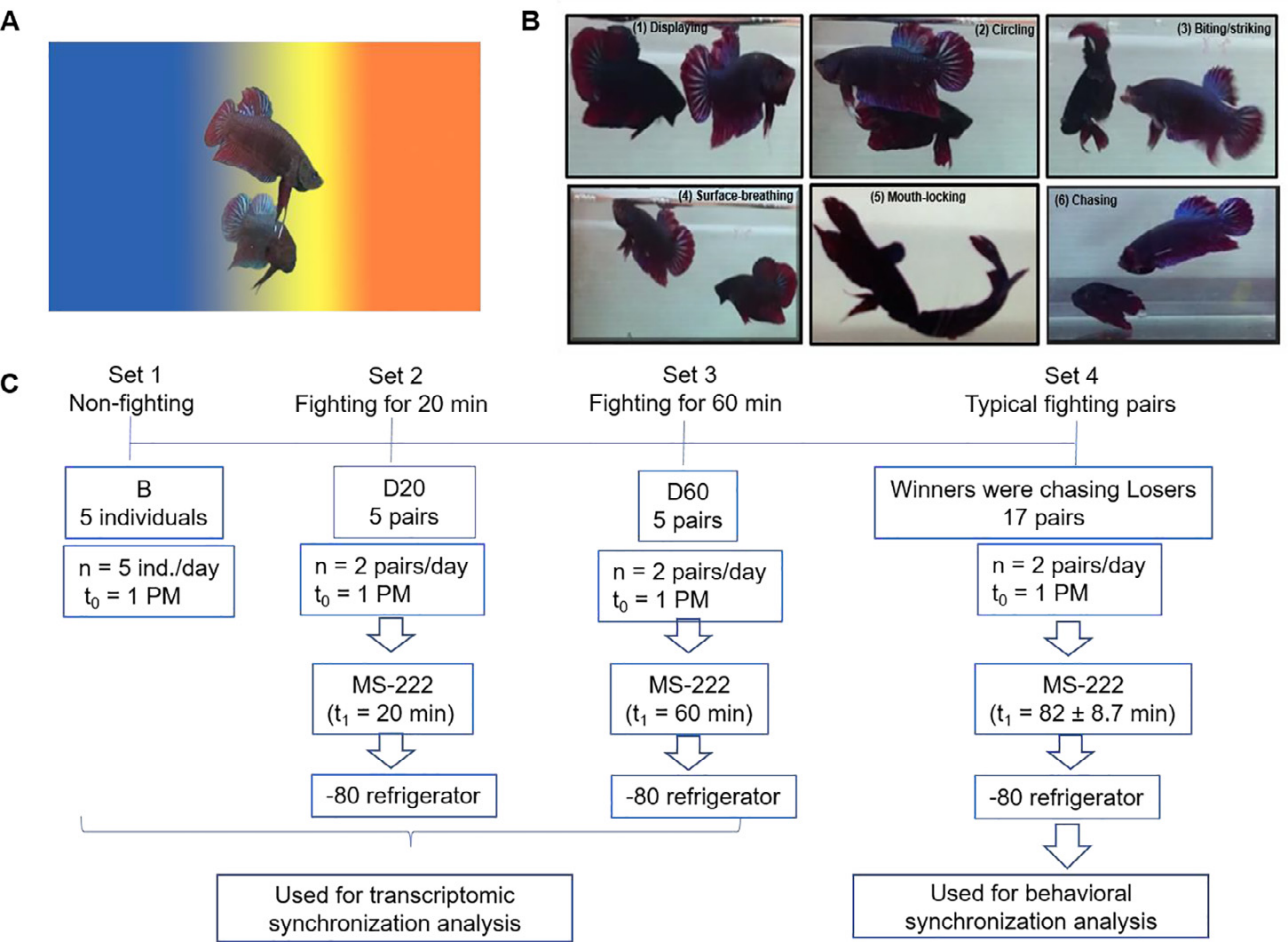
Dynamic fighting behaviors of two male *B. splendens* individuals (see 2.1.3 for details).

Several males of *B. splendens* (average standard length, 5.2 ± 1.1 cm) were imported from a local fish shop in Thailand. When they were brought to the laboratory for testing, all experimental males were isolated for at least one week. All fish were fed with commercial food daily and kept on a 12 h light/12 h dark cycle. The aggressive behaviors of this fish have been described previously [6,7]. For the behavioral test, briefly, several pairs of males *B. splendens* were introduced to fight each other in a small tank in a 1.7-L PVC tank (18 × 12.5 × 7.5 cm). Their fighting process took place in a sequence beginning with displaying behavior in which two individuals spread their fins and their body colors turned bright, next they circled to examine each other. Then, they bite/strike and went up to the surface to take oxygen (surface-breathing) or performed mouth-locking behaviors. Finally, one fish chased the other and this chasing period signified that the fight ended and the winner/loser became evident (Fig. 1A, B).

#### 2.1.2 The fighting sets

Five groups of fish collected from four experimental sets were analyzed (Fig. 1C): (i) non-fighting fish (B, *n* = 5 individuals; B1, B2, B3, B4, B5) were used as a control group and were not exposed to other fish; (ii) fish that were allowed to fight for 20 min (D20; *n =* 5 pairs; D20-11 vs. D20-12, D20-21 vs. D20-22, D20-31 vs. D20-32, D20-41 vs. D20-42, and D20-51 vs. D20-52); (iii) fish that were allowed to fight for 60 min (D60; *n =* 5 pairs; D60-11 vs. D60-12, D60-21 vs. D60-22, D60-31 vs. D60-32, D60-41 vs. D60-42, and D60-51 vs. D60-52); (iv) fish that were allowed to fight just until one fish chased the other (A0; *n =* 3 pairs; W0-1 vs. L0-1, W0-2 vs. L0-2, and W0-3 vs. L0-3), which takes 79.6 ± 9.8 min on average; and fish that were allowed to fight until one fish chased the other and then were collected 30 min later (A30; *n =* 3 pairs: W30-1 vs. L30-1, W30-2 vs. L30-2, and W30-3 vs. L30-3). We referred to the paired A0 fish as winners 0 (W0) and losers 0 (L0) and to the paired A30 fish as winners 30 (W30) and losers 30 (L30).

#### 2.1.3 Time of sample collection

Two fighting experiments (*n*) were conducted per day beginning at 1 PM (t0), and fish were immediately sacrificed at specific time points (t1) e.g., 20, 60 min, etc, by submersion in the lethal dose of MS 222. It took one day to collect the five individuals for Set1, three days to collect the five pairs for Set2, followed by another three days to collect the five pairs for Set3 as well as another 3 days for Set4. Brains for RNA-seq were collected before fighting (B, Set1), during fighting (D20 and D60, Set2 and Set3), and after fighting (A0 and A30, Set4). Their heads were frozen in liquid nitrogen, and their whole brains were carefully dissected and placed individually in Eppendorf tubes containing 1 mL of TRIzol Reagent (Life Technologies). After being sacrificed, the samples were immediately transferred to a −80 °C freezer and were stored there until subsequent brain dissection, RNA extraction, and RNA sequencing.

#### 2.1.4 RNA extraction

Total RNA was isolated using TRIzol Reagent according to the manufacturer's recommendation and was subsequently purified on columns with Quick-RNA MiniPrep (Zymo Research, USA). RNA was eluted in a total volume of 30 μL in RNase-free water. Samples were treated with DNase (QIAGEN) to remove genomic DNA. RNA quantity was assessed using a Qubit (Eugene, Oregon, USA), and RNA quality was assessed using the Agilent Bioanalyzer 2100 Nano kit (Agilent, USA) (RNA Integrity Number—RIN: 6.3–8.8). RNA was immediately stored at −80 °C until it was used to prepare the sequencing libraries.

#### 2.1.5 RNA-seq libraries preparation

RNA-seq libraries were constructed using the TruSeq Stranded mRNA Library Prep kit (Illumina, USA) with proper quality controls, and the molar concentrations were normalized using a KAPA Library Quantification kit (Kapa Biosystems, USA). Libraries were sequenced on the Illumina HiSeq 2500 system at Yourgene Bioscience Co., Ltd. (Taipei, Taiwan) and on the Illumina HiSeq 2500 system at the NGS High Throughput Genomics Core (Biodiversity Research Center, Academia Sinica, Taiwan).

### 2.2 Behavioral analysis

**Fig. 2:**
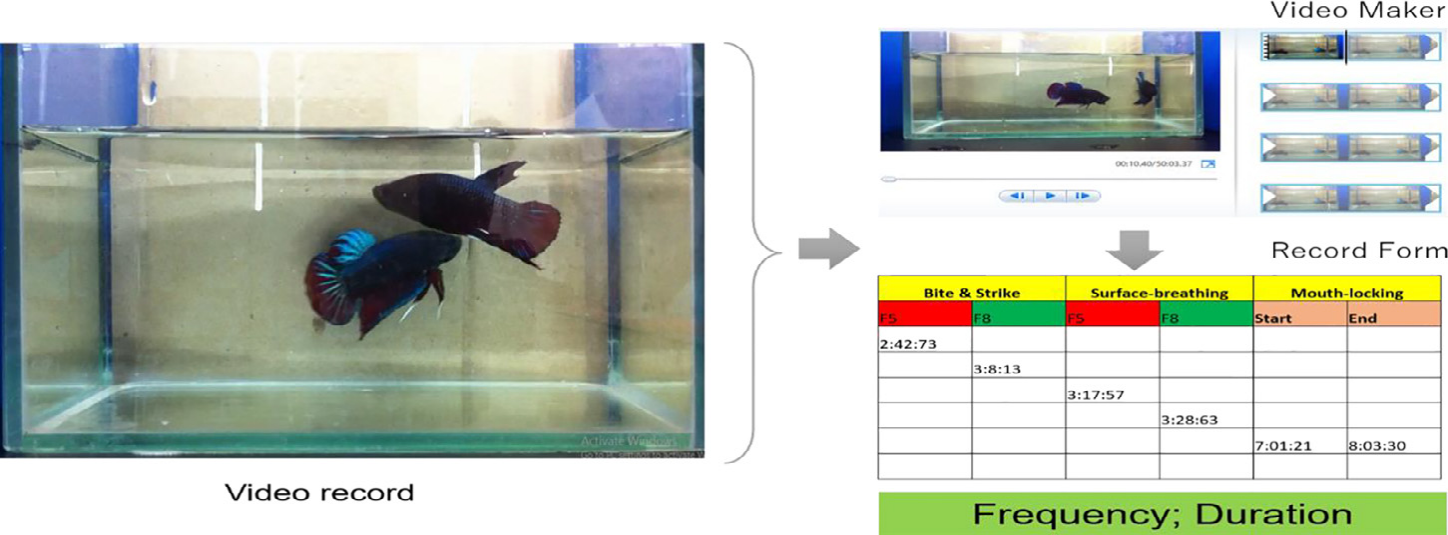
Behavioral analyses using video marker.

All the fighting pairs were videotaped by a camera (Nikon Cool Pix E5400). Then, the Video Marker tool was employed to tract the behavioral events e.g, biting/striking, surface-breathing, and mouth-locking for the behavioral analyses (Fig. 2). This tool allowed us to break down the time into minute: second: millisecond. Additionally, a record sheet was designed to mark all the behavioral events e.g., biting/striking, surface-breathing, and mouth-locking across the fighting process. Took the fighting pair F5 vs. F8 for an example, the fish F5 performed bite/strike first at 2:42:73 then the fish F8 performed this behavior at 3:8:13 as was shown. The other events for surface-breathing and mouth-locking could also be seen in the form. A total of seven fighting pairs was analyzed in terms of frequency and duration respecting each behavioral event in 80 min.

### 2.3 RNA-seq data analyses

FASTQC tool was used to assess the quality of the reads [8]. Adaptor sequences and low-quality bases were clipped from 50 bp single-end and paired-end sequences using the Cutadapt tool [9]. Reads were aligned to the *B. splendens* reference genome [10] using TopHat version 2.1.1 [11] and Bowtie2 version 2.1.0 [12] with the default settings. The unique mapping reads (reads that matched the reference genome at only one position) were extracted using Samtools [13]. The exon-mapped reads were counted with feature Counts [1]. The normalized expression levels of genes, represented by the trimmed mean of *M*-values (TMM), were generated with the edgeR [2] package in *R*.

### 2.4 Accession code

The RNA-Seq data are accessible on DDBJ (https://www.ddbj.nig.ac.jp/index-e.html) with these ID: DRA009599 and PRJDB11439

### 2.5 Statistical analysis

To define DEGs, we included genes with at least one count per million (cpm) in at least one sample. Count data were normalized by the TMM using edgeR in *R* [14]. To assess differential expression, a nested interaction model was fitted in edgeR. A tagwise dispersion estimate was used after computing common and trended dispersions. We adjusted the *p*-values from all contrasts at once concerning the false discovery rate (FDR). Two criteria were used to call DEGs: (i) the relaxed version used FDR < 0.05 alone, and (ii) a stringent version used both the FDR and FC value, with FDR < 0.05 and |logFC| > 2; these were implemented by the edgeR package in *R*.

The significantly enriched GO terms (biological process and molecular function terms) and KEGG pathways were identified by DAVID [15]. We tested for the overrepresentation of transcripts with a raw *p*-value of < 0.05 (Bayesian statistic).

## Ethics Statement

The animal experimentation procedures used in this study were approved by the Institutional Animal Care and Use Committee (IACUC) (Approval No. 106171) of the National Cheng Kung University, Tainan, Taiwan.

## CRediT authorship contribution statement

**Trieu-Duc Vu:** Data curation, Formal analysis, Validation, Writing – original draft, Writing – review & editing. **Yuki Iwasaki:** Data curation, Formal analysis, Validation. **Kenshiro Oshima:** Formal analysis, Writing – review & editing. **Ming-Tzu Chiu:** Formal analysis, Writing – review & editing. **Masato Nikaido:** Formal analysis, Writing – review & editing. **Norihiro Okada:** Conceptualization, Data curation, Formal analysis, Funding acquisition, Investigation, Methodology, Project administration, Supervision, Validation, Writing – original draft, Writing – review & editing.

## Declaration of Competing Interest

The authors declare that they have no known competing financial interests or personal relationships which have or could be perceived to have influenced the work reported in this article.

## Appendix Supplementary materials

Image, application 1
