# Supplementary material for: Data of RNA-seq transcriptomes in the brain associated with aggression in males of the fish Betta splendens
Source: Data Brief. 2021 Oct 2;38:107448. doi: 10.1016/j.dib.2021.107448 (PMC8503591; doi:10.1016/j.dib.2021.107448)
Supplement: Supplementary file 1 [file mmc1.pdf]

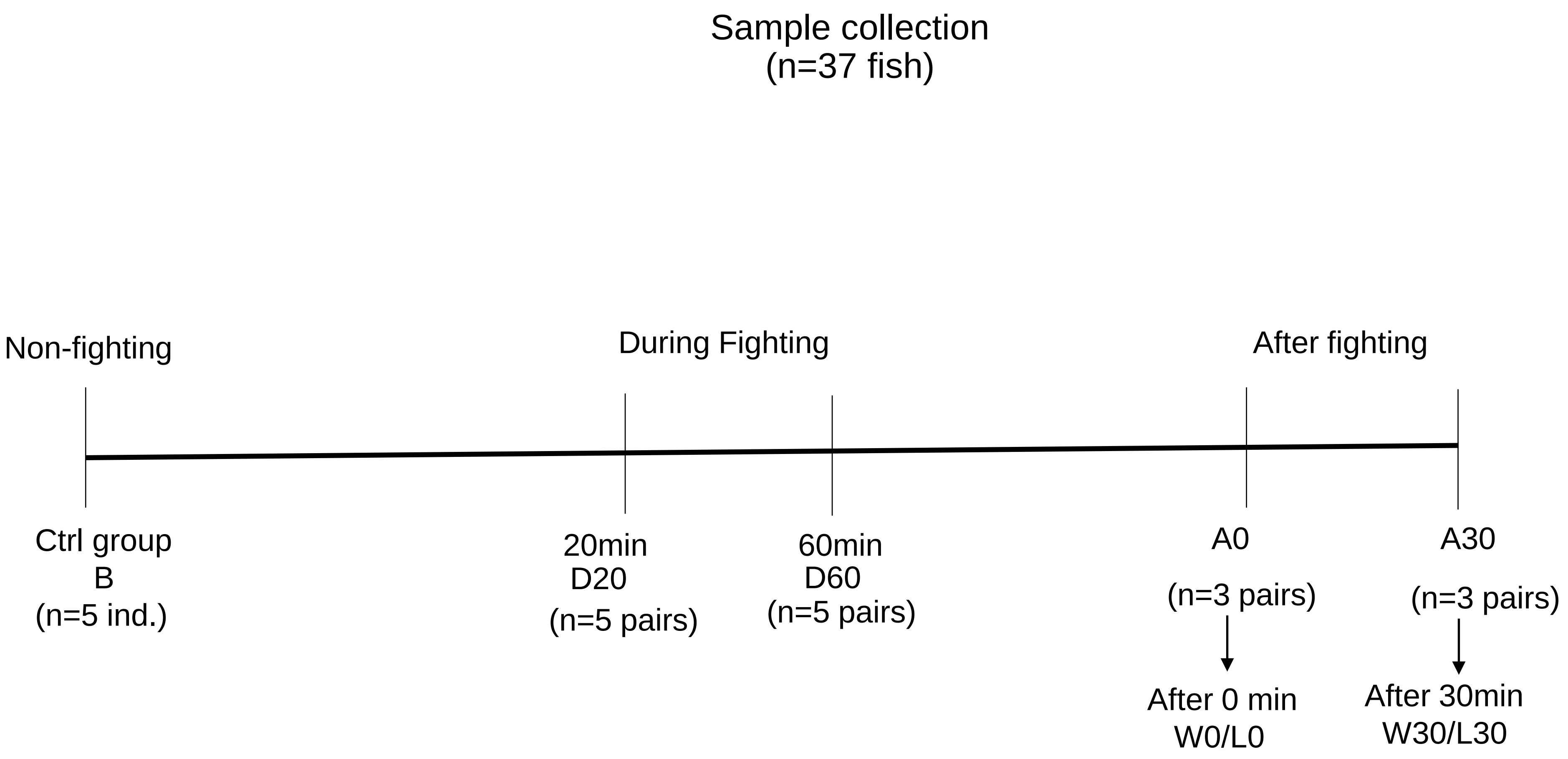

Fig. S1A. Experimental design. Thirty-seven samples were collected at different fighting durations. The samples included non-fighting (B), during fighting (D20 and D60), and after fighting (A0 and A30) fish. W0, winners collected at A0; L0, losers collected at A0; W30, winners collected at A30; L30, losers collected at A30

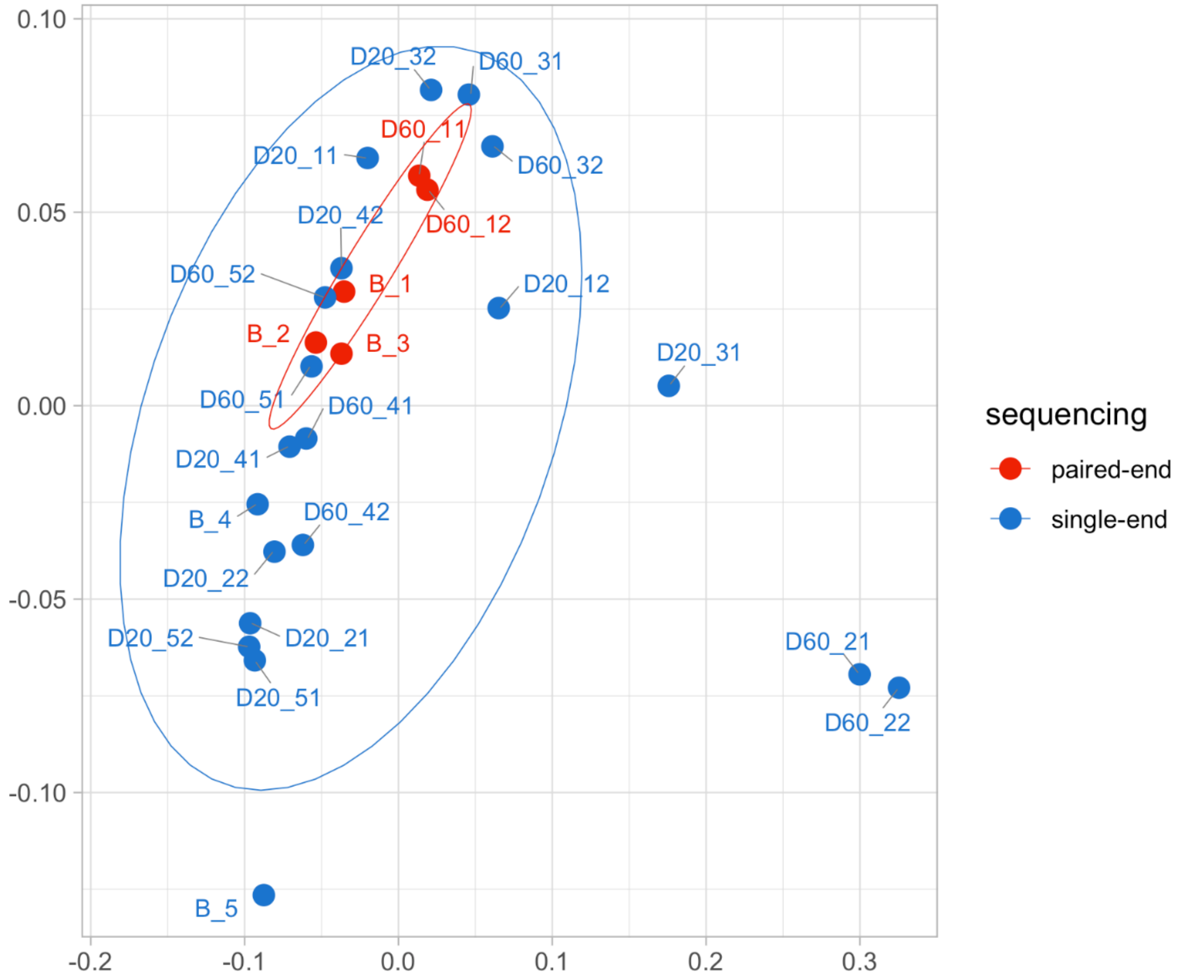

Fig. **S1B.** MDS plot confirming biases between single-end (blue) and paired-end (red) sequencing
